# Using Machine Learning to Predict Progression in the Gastric Precancerous Process in a Population from a Developing Country Who Underwent a Gastroscopy for Dyspeptic Symptoms

**DOI:** 10.1155/2019/8321942

**Published:** 2019-04-01

**Authors:** Susan Thapa, Lori A. Fischbach, Robert Delongchamp, Mohammed F. Faramawi, Mohammed S. Orloff

**Affiliations:** ^1^Department of Epidemiology, College of Public Health, University of Arkansas for Medical Sciences, Little Rock 72205, USA; ^2^Department of Biomedical Informatics, College of Medicine, University of Arkansas for Medical Sciences, Little Rock 72205, USA

## Abstract

**Background:**

Gastric cancer is the fourth most common cancer and the third most common cause of cancer deaths worldwide. Morbidity and mortality from gastric cancer may be decreased by identification of those that are at high risk for progression in the gastric precancerous process so that they can be monitored over time for early detection and implementation of preventive strategies.

**Method:**

Using machine learning, we developed prediction models for gastric precancerous progression in a population from a developing country with a high rate of gastric cancer who underwent gastroscopies for dyspeptic symptoms. In the data imputed for completeness, we divided the data into a training and a validation test set. Using the training set, we used the random forest method to rank potential predictors based on their predictive importance. Using predictors identified by the random forest method, we conducted best subset linear regressions with the leave-one-out cross-validation approach to select predictors for overall progression and progression to dysplasia or cancer. We validated the models in the test set using leave-one-out cross-validation.

**Results:**

We observed for all models that complete intestinal metaplasia and incomplete intestinal metaplasia were the strongest predictors for further progression in the precancerous process. We also observed that a diagnosis of no gastritis, superficial gastritis, or antral diffuse gastritis at baseline was a predictor of no progression in the gastric precancerous process. The sensitivities and specificities were 86% and 79% for the general model and 100% and 82% for the location-specific model, respectively.

**Conclusion:**

We developed prediction models to identify gastroscopy patients that are more likely to progress in the gastric precancerous process, among whom routine follow-up gastroscopies can be targeted to prevent gastric cancer. Future external validation is needed.

## 1. Introduction

In recent decades, rates of gastric cancer have declined in developed countries such as the United States [1, 2]. However, it has remained the fourth most common cancer globally, with nearly 1 million new cases diagnosed annually [1, 2]. In 2012, it was the third most frequent cause of cancer deaths worldwide [1, 2]. The prognosis for gastric cancer is highly dependent on the stage at which it is diagnosed; the 5-year relative survival rate remains as high as 70% for early-stage lesions and as low as 4% for advanced-stage lesions [3]. Therefore, routine monitoring for those at highest risk for progression to gastric cancer is important. Studies have reported that having undergone a prior gastroscopy before a gastric cancer diagnosis was associated with a lower mortality from gastric cancer [4–6]. Continued routine gastroscopies for those who are likely to progress to gastric cancer may further decrease mortality and improve survival by helping catch the disease at an early stage. However, in many cases, limited resources prevent the implementation of the continued routine gastroscopy programs, as most of these populations are located in the developing world where the burden of the disease is the greatest (150 per 100,000 in Nariño, Colombia, and 153 per 100,000 in men in Changle, China) [7–10]. A more efficient strategy to lower cost may be to identify and target individuals who are at high risk for progressing in the gastric precancerous process and are most likely to benefit from *H. pylori* treatment and routine gastroscopies.

Unfortunately, to date, the literature is limited with respect to factors that may facilitate progression to gastric precancerous lesions and/or cancer. The multistage etiologic model hypothesizes that a normal gastric mucosa progresses to one with nonatrophic gastritis, atrophic gastritis, intestinal metaplasia, then dysplasia, and gastric carcinoma in that order [11]. Approximately 65% to 80% of gastric dysplasias are reported to progress to gastric cancer [12]. Hence, identification of patients most likely to develop gastric dysplasia who can benefit the most from being routinely monitored for early identification of gastric cancer may help reduce mortality from gastric cancer. While causal effects of factors such as diet, smoking, family history, and *Helicobacter pylori* infection on the gastric precancerous progression have been investigated, there is scarcity of studies that have utilized machine learning to develop tools aimed at identifying patients more likely to progress in the gastric precancerous process. Hence, in this study, we used data from a 12-year cohort of participants in Nariño, Colombia, to develop tools to predict individuals who are most likely to develop the advanced precancerous lesions (dysplasia) or gastric cancer so that they can be targeted for routine monitoring more efficiently.

## 2. Materials and Methods

### 2.1. Study Population

We used data from an existing prospective cohort study that was conducted during 1993-2005 in Pasto, Colombia [7]. It consisted of a 16-week clinical trial consisting of the following five treatment regimens: (a) metronidazole, amoxicillin, and bismuth subsalicylate for the first two followed by bismuth alone for the next 14 weeks; (b) calcium carbonate (weeks 1-16); (c) treatment regimens (a) and (b); (d) tetracycline (weeks 1-16); or (e) placebo, followed by a short-term follow-up assessment (5 months after baseline) and a long-term follow-up assessment (11-12 years after baseline, in 2005) [7]. Study participants were residents of Pasto, Colombia, recruited mainly through community public service radio announcements, with a few through referrals made by local physicians [7]. Participants included in the study were ages 18 to 65 years, presented with symptoms of nonulcer dyspepsia, planned on continued residence within the Pasto city limits for at least 5 years, and provided informed consent [7]. Patients who took medications or had conditions likely to interfere with the trial medications (pregnancy, allergies to trial medications, etc.) were excluded [7]. Participants with baseline dysplasia, gastric cancer, peptic ulcers, or without *H. pylori* infection were excluded from the clinical trial but not from the long-term follow-up assessment [7]. Those who were not randomized to the anti-*H. pylori* therapy (the calcium, tetracycline, and placebo groups) were given the anti-*H. pylori* treatment or a prescription for the treatment after the trial ended at month 5.

### 2.2. Measurement of Gastric Precancerous Lesions

Our primary outcome of interest was any sequential progression in the gastric precancerous process. We measured progression in the gastric precancerous lesions by comparing histological diagnoses at 11-12-year follow-up to those at baseline. The histological diagnoses were obtained from gastric biopsies that were embedded in paraffin, sectioned, and stained with haematoxylin-eosin [7]. For the current analyses, biopsies were taken from the antrum and the corpus; an average of four biopsies at baseline and six biopsies at follow-up was taken from each patient. For each biopsy, the study pathologists provided independent histological reports; any discrepancies were resolved through discussions until the pathologists reached a consensus [7]. The updated Sydney System was used to classify and grade each biopsy for precancerous lesions [7, 13]. The Sydney System characterizes chronic nonatrophic gastritis, chronic atrophic gastritis, and intestinal metaplasia by the presence of mononuclear cells, the loss of glandular tissues, and the presence of goblet/absorptive cells (incomplete intestinal metaplasia) or colonocytes (complete intestinal metaplasia), respectively [13]. Gastric dysplasia is morphologically classified as intestinal (epithelial protrusion) and foveolar (irregular branching of glands and epithelial folding) [14].

### 2.3. Measurement of Potential Predictors of Progression in the Gastric Precancerous Lesions

We included a wide variety of potential predictors in the models. Broadly, we included demographics, dietary habits, medication use, anti-*H. pylori* treatment assigned during the clinical trial, biomarkers from urine analyses, and histological data in the analyses. The potential predictors are described below.

#### 2.3.1. Demographics, Dietary Factors, Medication Use, Urine Biomarkers, and Anti-*H. pylori* Treatments during the Randomized Clinical Trial Phase

The ages of the study participants were collected from their Colombian identification cards. Socioeconomic status was determined based on education, income, or car ownership collected at baseline and 5 months later based on participant interviews. Car ownership was likely to be a more accurate measure of socioeconomic status because only those in the high socioeconomic status owned cars. The interviews were also used to collect data on sex, smoking habits, alcohol consumption, the consumption of different food items (average weekly servings), and use of medications. During the interviews, participants were also measured for height and weight to estimate body mass index. Additionally, urine samples were taken from the eligible participants at baseline and 5 months to measure sodium, creatinine, calcium, and potassium levels as measures of their dietary intake. Creatinine correction was applied to urinary measures to account for urine volume and dilution. The sodium/creatinine ratio was used to estimate salt intake [15]. Since the literature on the effect of salt intake on gastric cancer progression is not homogeneous [16, 17], we used both self-reported and creatinine-corrected urinary sodium measures as predictors in our analyses. Creatinine-corrected urinary sodium estimates are the gold standard measures of dietary salt intake [18–20]. The treatments during the randomized trial ((a) metronidazole, amoxicillin, and bismuth subsalicylate; (b) calcium carbonate; (c) treatment regimens (a) and (b); (d) tetracycline; or (e) placebo) and eradication from the treatment were also investigated as predictors in the models.

#### 2.3.2. Histology

For this analysis, we used measures of the density of polymorphonuclear leucocytes, mononuclear leucocytes, loss of glandular tissues in the gastric mucosa, depth of the inflammation, and the density of *H. pylori* infection measured from the biopsies collected at baseline and 5 months. The density of polymorphonuclear leucocytes, mononuclear leucocytes, loss of glandular tissues in the gastric mucosa, and the density of *H. pylori* infection were scored from 0-3, where 3 was the highest density of inflammatory cells or *H. pylori* infection. We used average and maximum measures across biopsies. Histological assessment was conducted independently by three study pathologists. Any discrepancies in the histological diagnoses were resolved through discussions among the pathologists until a consensus was reached. We also studied presence and types of histological diagnoses at baseline as predictors. The histological diagnoses at baseline studied as potential predictors were no gastritis, nonatrophic gastritis (superficial gastritis and antral diffuse gastritis), gastritis with a diagnosis less than atrophic gastritis (no gastritis or nonatrophic gastritis), atrophic gastritis, and intestinal metaplasia. We subdivided intestinal metaplasia into complete (where gastric epithelial cells are replaced by columnar cells similar to the small intestine) and incomplete (where gastric epithelial cells are replaced by colonocytes or cells resembling the lining of the large intestine) types. This was done because complete intestinal metaplasia and incomplete intestinal metaplasia are two subtypes of intestinal metaplasia that show marked differences in their association with gastrointestinal diseases [21].

### 2.4. Data Analysis

The predictive modelling for gastric precancerous progression was conducted as described in the steps for the below following sets of variables: (1) all predictors, including those more common in the local population, and (2) more universally collected variables for patients undergoing a gastroscopy. We excluded data obtained from the urine sample and dietary factors uncommon outside of the local region for the more universally collected variables for patients undergoing a gastroscopy. We also ran models forcing in *H. pylori* eradication, as it is a putative strong risk factor for gastric cancer [1].

#### 2.4.1. Step I: Imputation

The first step in our analysis prior to predictive modelling was to conduct a regression/stochastic imputation of the missing data, wherein the distribution of the observed data was used to predict the missing values [22].

#### 2.4.2. Step II: Training and Test Datasets

The complete imputed dataset was then randomly divided into a training set (80% *n* = 247), in which the prediction model was to be developed, and a test set (20%, *n* = 61), in which the developed model was to be validated. The standard Pareto Principle with an 80%/20% split was used [23]. We also obtained training and test sets using randomly divided 50%/50% and 63.2%/36.8% splits [24]. (We ultimately kept the sets with the best prediction in Step V.)

#### 2.4.3. Step III: Variable Importance

The training set had over 100 covariates. Hence, we first prioritized the potential predictors to be included in the predictive models. This was done to ensure the inclusion of predictors with the highest predictive importance in the best subset linear regression for variable selection that only allows for 30 or fewer variables to be included in each model. Prioritization of the predictors was done by ranking the predictors based on their predictive importance (variable importance). Variable importance was estimated based on the use of variables in the final decision tree (i.e., using the random forest approach). We used relative importance to rank the predictors. The relative importance measures were based on the changes in sums of square error (SSE) for each variable because of the split in the decision tree. A value of higher importance was assigned to each variable relative to the maximum SSE.

#### 2.4.4. Step IV: Predictive Modelling Using Best Subset Regression in the Training Set

Variables identified by the random forest as having high predictive importance were included in models for prediction of progression in the gastric precancerous process from baseline to 11-12-years of follow-up. We used best subset linear regression leaving one observation out in each iteration to select the predictors in the training set. Using the best subset regression with leave-one-out cross-validation, one observation was left out in each iteration of the regression, such that *n* − 1 observations were used for each iteration of the regression. The subsets of predictors that best explained the data were then identified based on a *R*^2^.

#### 2.4.5. Step V: Validation Using the Test Set

The models developed from the dataset using all variables and the dataset restricted to more universally collect data (with and without forcing *H. pylori* eradication at 5 months into the model) were then validated using the 20% test (or validation) sample. We used the leave-one-out cross-validation method to obtain the predicted probabilities for the outcome [25]. We used the median as a cut-off for classifying the predicted probabilities for the outcomes into the group that progressed (≥median) and the group that did not progress (<median). We then estimated the sensitivity and the specificity of the identified models in predicting the progression to gastric precancerous process in the test set, using change in the available histological diagnoses overtime as the gold standard.

**Figure 1:**
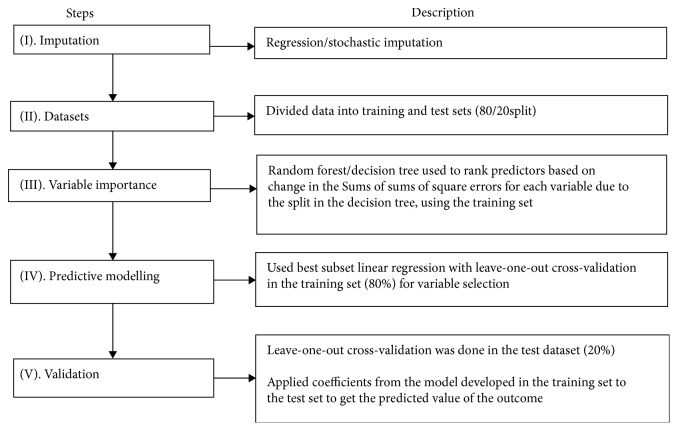
Steps of data analyses for prediction of gastric precancerous progression.

The steps of the data analyses are summarized in Figure 1. Two models were developed using these steps: (a) a location-specific model to predict overall progression in the gastric precancerous process, where all variables were included, and (b) a general model to predict overall progression in the gastric precancerous process, where variables specific to the study location were excluded to increase generalizability and utility of the model.

## 3. Results and Discussion

### 3.1. Results

Our study sample consisted of 308 individuals. Overall, 127 participants progressed in the gastric precancerous process during the 11-12-year follow-up compared to baseline, including 13 participants who progressed to dysplasia or gastric cancer.

The prediction models showed greater accuracy for the set divided into 80% for the training set and 20% for the test set; therefore, we are only reporting these prediction models. The random forest method for variable importance identified 20 predictors using the dataset with all variables, and 13 predictors for the more universally collected variables that had the highest predictive importance. The ranked list of predictors with the most predictive values based on SSE for each outcome is listed in Table 1.

We conducted the best subset linear regression with leave-one-out cross-validation to identify the best models for predicting the overall precancerous progression. The predictors listed in Table 1, identified by random forest, were included in the best subset predictor models for each outcome. The final set of predictors identified by each model and the *R*-squared values are summarized in Table 2. The sensitivity and the specificity of the general model in predicting overall progression were 86% and 79%, respectively. Similarly, the sensitivity and the specificity of the location-specific model in predicting overall progression were 100% and 82%, respectively. The sensitivity and specificity for each model are listed in Table 2. The addition of *H. pylori* eradication did not improve the sensitivity or specificity of either predictor model.

Table 3 shows that complete intestinal metaplasia and incomplete intestinal metaplasia were the most important predictors used for overall progression in the gastric precancerous process; this was observed for all models including all models using the 50%/50% and the 63.2%/36.8% splits. Additionally, those with atrophic gastritis at baseline progressed more than those with diffuse antral gastritis, superficial gastritis, and no gastritis at baseline in all models, and those who had deeper corpus inflammation were less likely to progress compared to those with more superficial inflammation.

In the dataset with all variables, a higher intake of fried fava beans per week at baseline as well as higher baseline density of *H. pylori* infection that was present in both the corpus and the antrum at baseline increased the overall progression (Table 3). In the dataset with more commonly collected variables for gastroscopy patients, alcohol intake and average density of polymorphonuclear cells in the antrum at baseline were identified as predictors.

### 3.2. Discussion

In this study, we developed models to predict overall progression in the gastric precancerous process in a high-risk population. In the cohort, over a third of the participants showed progression in the precancerous process.

It is important to note that regression coefficients obtained from the prediction models (Table 3) may not reflect causal associations of the predictors with the outcomes of interest because these coefficients are not obtained from a causal model. A causal model estimates the association between an exposure and an outcome adjusting only for covariates that are potential confounders to get an unbiased estimate of the effect of the exposure on the outcome, whereas, a prediction model allows for inclusion of any covariate, including an intermediate, in the model that may predict the outcome [26]. Hence, the identified predictors in our models may not necessarily be causal risk factors for gastric precancerous progression. Nevertheless, a factor that is causally associated with the outcome or an intermediate is more likely to predict the outcome compared to other factors. Hence, factors with a causal association with gastric cancer are more likely to be identified as predictors in the prediction models and this was observed in our results for *Helicobacter pylori* infection-induced inflammation.

We found that the presence of complete or incomplete intestinal metaplasia was a predictor for gastric precancerous progression in all models. This is consistent with previous studies. Intestinal metaplasia has been associated with progression to dysplasia or gastric cancer, with no reversal in progression even after administration of interventions such as *H. pylori* eradication therapies that have been efficacious in reversing early-stage precancerous lesions like atrophic gastritis [10, 27, 28].

Similarly, higher density of *H. pylori* infection at baseline when present in both the corpus and the antrum or density of inflammatory cells was a predictor of overall gastric precancerous progression in our analyses. This is consistent with the existing literature that has reported *H. pylori* to be a class I carcinogen [29] that may play an important role in progression from a normal gastric mucosa to gastric precancerous lesions and gastric cancer by eliciting an inflammatory response [8, 30]. In our study, infection present in both the corpus and the antrum may have been a predictor possibly because *H. pylori* infection usually begins in the pyloric region and then extends to the corpus [31]. Hence, *H. pylori* infection present in both the corpus and the antrum regions may be an indication of more advanced and widespread *H. pylori*-induced inflammation and may predict gastric precancerous progression.

Higher weekly intake of fried fava bean, which is a food item consumed in Nariño, Colombia, was another predictor of overall progression in the models using variables specific to the local population. In Colombia, intake of fried fava beans has been thought to contribute to gastric cancer. A Colombian nutritional survey has also reported an association between fava bean intake and gastric cancer [32]. Fried fava beans may have higher polycyclic aromatic hydrocarbon content due to their burnt nature. Polycyclic aromatic hydrocarbons have been reported to increase cancers of the other area of the body and may also increase gastric carcinogenesis [33]. Our current results were consistent with the existing literature in that higher weekly intake of fried fava beans in Colombia was associated with an increased overall progression in the gastric precancerous process.

Our study has several strengths. First of all, our study is one of the very few studies that have conducted prediction modelling for gastric cancer progression. A previous study which developed a predictor model focused on identifying high-risk groups using covariates collected from questionnaires among patients who had not undergone prior gastroscopies in Korea [34]. This previous gastric cancer prediction model identified the following predictors: age, body mass index, family history of gastric cancer, meal regularity, salt preference, alcohol consumption, smoking and physical activity for men, and age, body mass index, family history, salt preference, alcohol consumption, and smoking for women [34]. Our models considered similar predictors—family history of gastric cancer, body mass index, age, alcohol intake, salt intake, and smoking. However, unlike the Korean model, we also considered important characteristics such as *H. pylori* infection, density of inflammation, and presence of precancerous lesion, obtained from histological assessment of gastroscopic biopsies, and these were observed to be stronger predictors. Another predictor model, the ABCD screening method, has been used in Japan for prediction of gastric cancer risk [35]. This method differs from our models in that it uses measures of serum IgG anti-*Helicobacter pylori* antibody and serum pepsinogen [35]. Our study is unique in that it is geared towards patients who have undergone a gastroscopy. Additionally, our model is consistent with the management of precancerous conditions and lesions in the stomach (MAPS) guideline, which emphasizes surveillance for patients with more advanced histological diagnoses [36].

Additionally, we used multiple methods to predict gastric precancerous progression. The random forest/decision tree method to identify factors with the greatest prediction importance followed by best subset regression and leave-one-out cross-validation approaches for selection of predictors and cross-validation of the selected models, also known as machine learning, is a powerful tool compared to traditional statistics when large numbers of covariates are available. Machine learning approaches such as random forest/decision trees and artificial neural networks use interconnected graphical models for predicting an outcome [37]. Random forests and artificial neural networks have become increasingly popular in medical research, particularly for prediction of cancers [37]. These techniques have an advantage over traditional statistics in that they can identify implicit interactions when present and can be more powerful in predicting the outcomes.

Our study is not without limitations. Our study had a small sample size of 308 individuals, which may affect the validity of our study. Sensitivity and specificity were estimated on a validation set of 61, which is a relatively small sample. More robust results can be achieved with a larger dataset. Additionally, our study population in Colombia may differ substantially from populations in developed countries. As mentioned previously, the study was conducted in Nariño, Colombia, which has one of the highest reported rates of gastric cancer [8]. We did not observe *H. pylori* eradication to be a strong predictor in our population. This may be due to the observation that treatments for *H. pylori* infection tend to be less effective and reinfection rates tend to be much high in developing countries [38]. Therefore, our predictor models may not be generalizable to population in countries where *H. pylori* treatments are more successful or where reinfection of the bacterium is rare. Thus, like other predictor models in the field, external validation in a different study cohort was not done to test the generalizability of the models.

## 4. Conclusions

Identification of gastroscopy patients who are likely to progress to gastric precancerous lesion or gastric cancer is important because this high-risk group can be targeted with routine follow-up gastroscopies for implementation of preventive strategies and early cancer detection. Furthermore, routine follow-up gastroscopies only among those that are more likely to progress in the precancerous process is a more cost-effective approach compared to population screening for developing countries, where the burden of the disease is the greatest. Our study provides predictors to identify individuals most likely to progress to gastric precancerous lesions or cancer in the future so that they can be targeted with routine gastroscopies for preventive strategies and routine monitoring. These prediction models should be externally validated for better assessment of their predictive performances.

## Figures and Tables

**Table 1:** Predictors ranked (variable importance) using random forest method based on sums of square error (SSE) in the training set.

Variables ranked	Relative importance
*Overall progression (general model)*	
Complete intestinal metaplasia at baseline	1
Diagnosis at baseline less advanced than atrophic gastritis	0.80368
Incomplete intestinal metaplasia at baseline	0.64445
Depth of corpus inflammation at baseline	0.43734
Alcohol intake at baseline (yes/no)	0.39928
Total loss of glandular tissue at 5 months	0.30324
Received anti-*H. pylori* treatment	0.28874
Average density of polymorphonuclear cells in the antrum at baseline	0.26655
Body mass index	0.26324
Education level at baseline	0.25006
Average density of polymorphonuclear cells in the corpus at baseline	0.22872
Years of intake of proton pump inhibitors at baseline	0.22002
Total depth of inflammation at baseline	0.20417
*Overall progression (location-specific model)*	
Complete intestinal metaplasia at baseline	1
Diagnosis at baseline less advanced than atrophic gastritis	0.72046
Incomplete intestinal metaplasia at baseline	0.63149
Urinary calcium at baseline	0.43548
Total loss of glandular tissue at 5 months	0.41663
Depth of corpus inflammation at baseline	0.40250
Depth of total inflammation from antrum/corpus at baseline	0.34093
Bloating at baseline	0.31531
Average density of polymorphonuclear cells in the corpus at baseline	0.31494
Intake of coffee with milk at baseline	0.2875
Total mucus depletion at baseline	0.2635
Alcohol intake at baseline	0.25981
Years of use of proton pump inhibitors	0.25814
Intake of fried fava beans at baseline	0.21631
Use of analgesics at baseline	0.21515
Depth of total inflammation from antrum/corpus at 5 months	0.18031
Antacid use at baseline	0.17009
Density of *H. pylori* infection in the corpus and the antrum at baseline	0.16174
Any reflux at baseline	0.15910
Intake of fruits and vegetables at baseline	0.10299

**Table 2:** Predictors identified based on the best subset regression with the leave-one-out method and model validation using leave-one-out cross-validation.

Outcome	Best subset model with the leave-one-out method		Validation of model identified by best subset with the leave-one-out method in the 20% test sample	
	Identified predictors	*R* ^2^	Sensitivity (%) (*a*/*a* + *b*)	Specificity (%) (*d*/*c* + *d*)
Overall progression (general model)	Complete intestinal metaplasia	41.0	86 (19/22)	79 (31/39)
	Incomplete intestinal metaplasia			
	Histological diagnosis at baseline less advanced than atrophic gastritis			
	Depth of corpus inflammation at baseline			
	Average density of polymorphonuclear cells in the antrum at baseline			
	Alcohol intake at baseline			

Overall progression (location-specific model)	Complete intestinal metaplasia	43.1	100 (21/21)	82.1 (32/39)
	Incomplete intestinal metaplasia			
	Histological diagnosis at baseline less advanced than atrophic gastritis			
	Average density of *H. pylori* infection in the corpus and the antrum			
	Depth of corpus inflammation at baseline			
	Intake of fried fava beans			

^∗^Sensitivity and specificity were identical when eradication of H. pylori infection was forced into the model.

**Table 3:** Results of linear regression for the predictors identified by best subset linear regression with the leave-one-out method.

Outcome	Predictors	Regression coefficients (95% confidence intervals)
Overall progression (general model)	Complete intestinal metaplasia	0.534 (0.425, 0.644)
	Incomplete intestinal metaplasia	0.316 (0.187, 0.444)
	Histological diagnosis at baseline less advanced than atrophic gastritis	-0.313 (-0.368, -0.258)
	Depth of corpus inflammation at baseline	-0.152 (-0.265, -0.039)
	Average density of polymorphonuclear cells in the antrum at baseline	0.037 (-0.029, 0.103)
	Alcohol intake at baseline	-0.189 (-0.201, 0.022)

Overall progression (location-specific model)	Complete intestinal metaplasia at baseline	0.492 (0.382, 0.602)
	Incomplete intestinal metaplasia at baseline	0.345 (0.223, 0.467)
	Histological diagnosis at baseline less advanced than atrophic gastritis	-0.296 (-0.351, -0.241)
	Depth of corpus inflammation at baseline	-0.150 (-0.264, -0.037)
	Density of *H. pylori* infection in the corpus and the antrum at baseline	0.122 (0.030, 0.214)
	Intake of fried fava beans per week at baseline	0.064 (0.0004, 0.128)

## Data Availability

Data is not available.
